# Structure and Dynamics of Supramolecular Polymers: Wait and See

**DOI:** 10.1021/acsmacrolett.2c00223

**Published:** 2022-05-09

**Authors:** Sandra M. C. Schoenmakers, A. J. H. Spiering, Svenja Herziger, Christoph Böttcher, Rainer Haag, Anja R. A. Palmans, E. W. Meijer

**Affiliations:** † Laboratory of Macromolecular and Organic Chemistry, Institute for Complex Molecular Systems, Eindhoven University of Technology, 5612 AZ Eindhoven, The Netherlands; ‡ Institute of Chemistry and Biochemistry, Freie Universität Berlin, 14195 Berlin, Germany; § Research Center of Electron Microscopy and Core Facility BioSupraMol, Institute of Chemistry and Biochemistry, Freie Universität Berlin, 14195 Berlin, Germany; ∥ School of Chemistry, University of New South Wales, Sydney NSW 2052, Australia

## Abstract

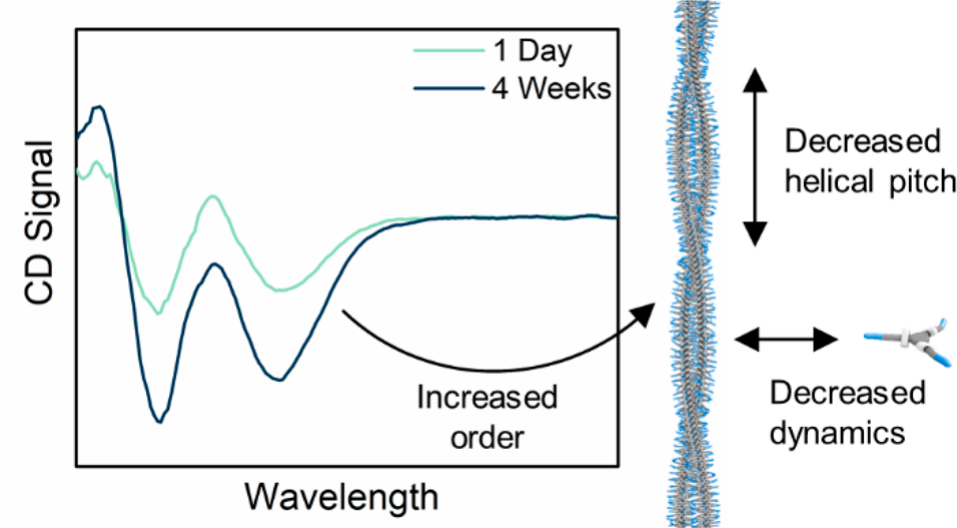

The introduction of stereogenic centers in supramolecular building blocks is used to unveil subtle changes in supramolecular structure and dynamics over time. Three stereogenic centers based on deuterium atoms were introduced in the side chains of a benzene-1,3,5-tricarboxamide (BTA) resulting in a supramolecular polymer in water that at first glance has a structure and dynamics identical to its achiral counterpart. Using three different techniques, the properties of the double helical polymers are compared after 1 day and 4 weeks. An increase in helical preference is observed over time as well as a decrease in the helical pitch and monomer exchange dynamics. It is proposed that the polymer of the chiral monomer needs time to arrive at its maximal preference in helical bias. These results indicate that the order and tight packing increase over time, while the dynamics of this supramolecular polymer decrease over time, an effect that is typically overlooked but unveiled by the isotopic chirality.

Natural supramolecular systems exhibit a wide range of structures and dynamics, sometimes even within one assembly. The amino acid sequences in proteins that lack a secondary structure show more fluctuations in space than amino acid sequences contained in α-helices or β-sheets.^1−3^ Similarly, the amphiphilic phospholipids in the cell membrane are limited in their lateral diffusion when tightly packed, for example when they are assembled into lipid rafts.^4−6^ The structure, dynamics, and function of those assemblies are influenced by the order within the assemblies: the tighter the packing of the molecules, the less dynamic they are. Likewise, synthetic supramolecular assemblies made from small amphiphiles can show different structures^7−9^ and dynamics^10^ depending on the packing of the monomers. Gaining control over the order within supramolecular assemblies will help to gain control over their properties. Moreover, when the dynamics are slow, it will take a long time before the structure will be in its thermodynamically most favorable state, an issue often overlooked.

Synthetic supramolecular polymers in water are promising candidates for the formation of synthetic biomaterials since they share a lot of similarities in their structure and dynamics to living tissues.^11−15^ Several types of supramolecular polymers have been designed for this purpose, based on various motifs such as peptide amphiphiles,^16−18^ ureidopyrimidinones,^19,20^ or discotics.^21−24^ Recent work of Stupp et al. disclosed the importance of dynamicity in the peptide amphiphiles for the biological impact of these biomaterials.^25^ Our group has focused on water-compatible supramolecular polymers based on benzene-1,3,5-tricarboxamides (BTAs) with a dodecyl spacer attached to the amides and a tetra(ethylene glycol) periphery for compatibilization with water (**BTA-C**_**12**_**-EG**_**4**_, Chart 1).^26^ Supramolecular monomers can easily be modified, and even the smallest modifications can result in supramolecular polymers with drastically different properties.^24,25,27−34^ A major challenge lies in the ability to predict how small changes in the chemical structure of the monomers affect molecular packing, the overall structure, and the exchange dynamics of the supramolecular polymers. These properties of the supramolecular polymers are generally studied over a short time period, as these polymers are assumed to reach thermodynamic equilibrium in a reasonably short period of time.^10,23,28,35,36^ Surprisingly, hydrogen/deuterium exchange experiments used to elucidate the exchange dynamics of BTAs indicate that some monomer exchange processes occur within minutes, while others occur over longer time scales, even in the range of weeks.^37^ This broad spectrum of dynamics is even observed in the same sample. Additionally, detailed microscopy studies revealed the presence of a secondary double helical structure within those polymers that was previously overlooked.^38^ An understanding of the arrangement at the molecular level is therefore essential to understand these properties of the systems at the supramolecular level.^28^

**Chart 1:**
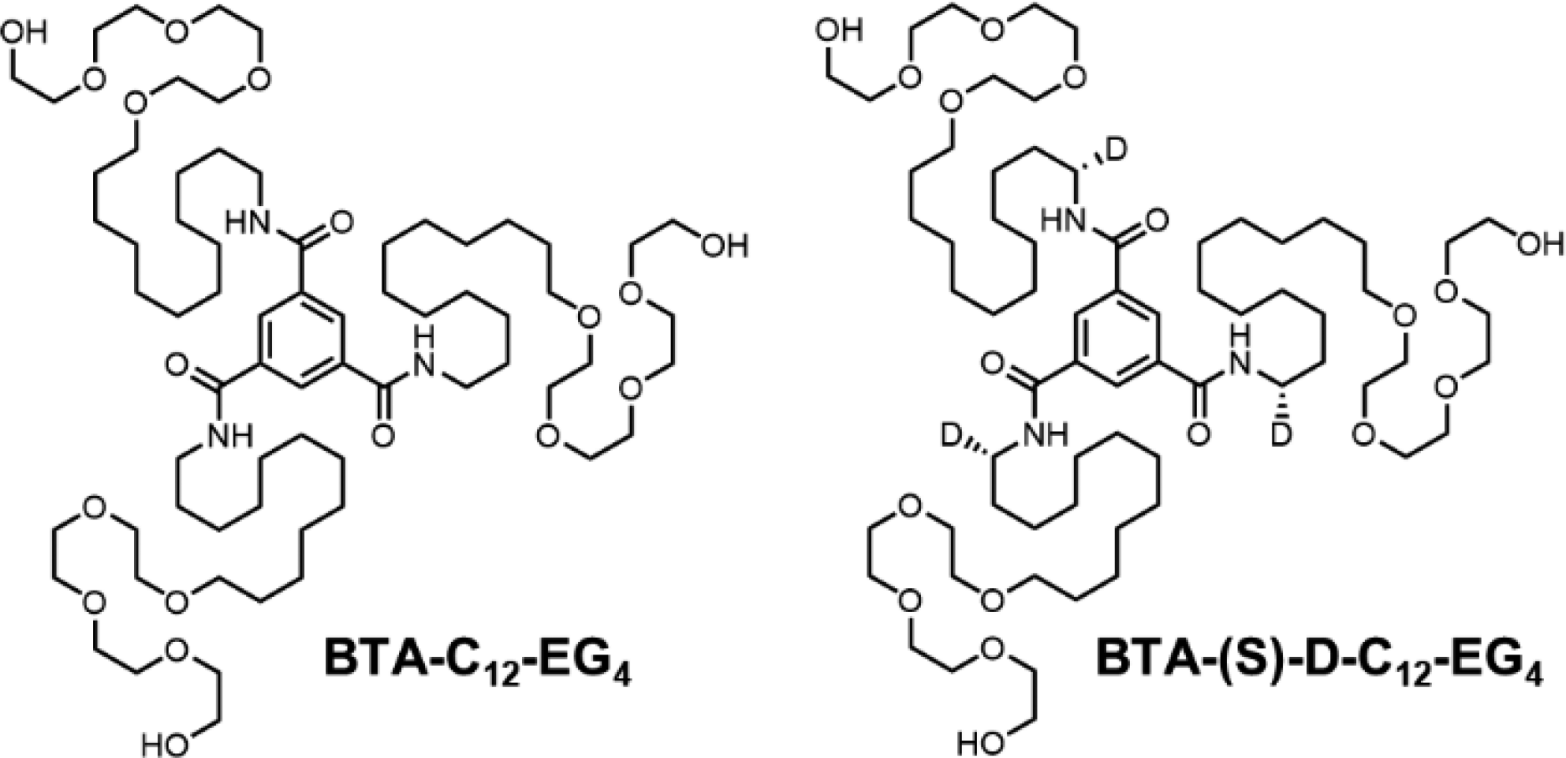
Chemical Structure of Achiral **BTA-C**_**12**_**-EG**_**4**_ and Chiral **BTA-(S)-D-C**_**12**_**-EG**_**4**_

One-dimensional supramolecular polymers assembled from discotic building blocks, such as BTAs, are characterized by their helical arrangement, which can be biased by the introduction of stereogenic centers in the monomers.^39^ This has already led to several new revelations about the self-assembly of supramolecular polymers in organic solvents.^40−43^ Hydrogen/deuterium substitution, not related to hydrogen/deuterium exchange experiments to study dynamics mentioned above, introduces stereogenic centers with only a minimal chemical modification, while the zero-point energy difference between the C–H and C–D stretch next to an amide is enough to induce a helical preference.^44,45^ The introduction of a deuterium atom at the α-position of the side chains of BTAs resulted in the formation of supramolecular polymers with a preferred helicity in organic solvents.^43^ The isotopic chirality helped to elucidate conformational changes due to solvent and temperature effects in those chiral polymers.^44,46^ The introduction of chirality is rarely explored for supramolecular polymers in water as the introduction of bulky stereogenic groups changes the delicate hydrophilic/hydrophobic balance, thereby altering their packing or even their ability to form elongated structures.^26,38,47^

Here, we use isotope chirality to gain a better understanding on the self-assembly of BTA-based supramolecular monomers in water. **BTA-(S)-D-C**_**12**_**-EG**_**4**_ (Chart 1) was previously shown to assemble into supramolecular polymers with a Cotton effect that increases over time.^37^ The effect of this increase is studied in detail in relation to an increase in order on the supramolecular structure and on the exchange dynamics using different techniques.

Deuterium atoms were stereoselectively introduced on the α-position next to the BTA amides via an enzymatic reduction using the alcohol dehydrogenase *Thermoanaerobacter* sp. (ADH-T) and isopropanol-*d*_8_ as a deuterium source (see Supporting Information, sections 2 and 3 for more details). The stereoselectivity of the reduction was found to be very high, with an enantiomeric excess >95%. Our previously optimized self-assembly protocol was used to obtain hydrogen-bonded supramolecular polymers in water with spectroscopic features identical to that of **BTA-C**_**12**_**-EG**_**4**_ (Figures S1 and S2), thereby confirming that the packing did not change by the introduction of the deuterium atoms.

The circular dichroism (CD) spectrum of **BTA-(S)-D-C**_**12**_**-EG**_**4**_ is characterized by a positive Cotton effect at 196 nm and negative Cotton effects at 213 and 250 nm (Figure 1). The occurrence of a CD signal indicates that an ordered packing with a preferred helicity is adopted. This effect is weak due to the small mismatch penalty between hydrogen and deuterium atoms.^44−46^ The slightly positive band at 230 nm overlaps with a negative linear dichroism (LD) signal (Figure S3), indicating that this band originates from macroscopic alignment of the polymers in the cuvette rather than a helical bias.^48^ Interestingly, the UV spectrum is unaffected by time whereas the CD signal changes over time. The Cotton effects at 213 and 250 nm double in intensity over 4 weeks, whereas the LD signal diminishes, with the most prominent changes observable in the first 2 weeks (Figure 1). Only after the equilibration time, the intensity of the CD signal is similar to that of an alkane-soluble BTA with isotopic chirality after 1 day in an organic solvent.^43,46^ The time-dependent increase in CD signal indicates a slow increase of helical order, which is independent of concentration (Figure S4). No amplification of asymmetry was observed when **BTA-(S)-D-C**_**12**_**-EG**_**4**_ was mixed with **BTA-C**_**12**_**-EG**_**4**_ in a so-called Sergeant-and-Soldier experiment (Figure S5), which is likely caused by a low mismatch penalty.^45^

**Figure 1:**
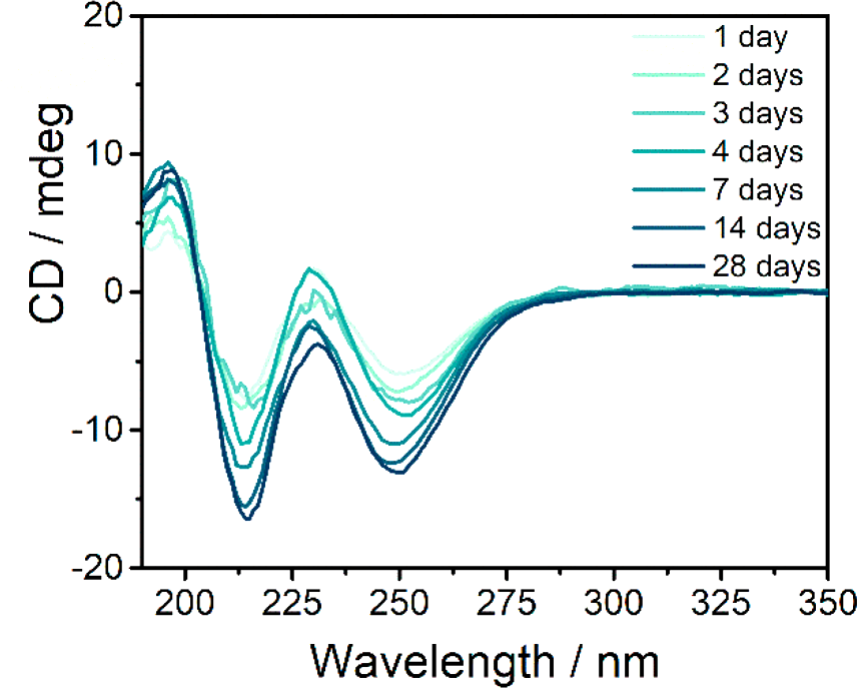
CD spectra of **BTA-(S)-D-C**_**12**_**-EG**_**4**_ (*c* = 50 μM, *l* = 1 cm, *T* = 20 °C) in water over time.

In order to explain the increase in optical activity, the morphologies formed by **BTA-(S)-D-C**_**12**_**-EG**_**4**_ in water were visualized with cryogenic transmission electron microscopy (cryoTEM). Micrometer long one-dimensional polymers are observed with a diameter of around 7 nm (Figures 2A and S6). Image processing techniques previously used to analyze the secondary structure of BTA-based polymers were applied to further examine the polymers of **BTA-(S)-D-C**_**12**_**-EG**_**4**_.^38^ A double helix structure with a half pitch of 19.9 ± 0.4 nm (Figure 2B) was found after 1 day of equilibration, which is identical to the secondary structure of **BTA-C**_**12**_**-EG**_**4**_. The image extracts show only a half pitch and multivariate statistical analysis was used to confirm that the helical pattern is repeated uniformly over the length of the supramolecular polymers. After 4 weeks of equilibration, the half helical pitch decreased to 18.6 ± 0.4 nm (Figure 2C), indicative for a tighter packing of molecules. This nicely coincides with the increase in order as observed with CD spectroscopy.

**Figure 2:**
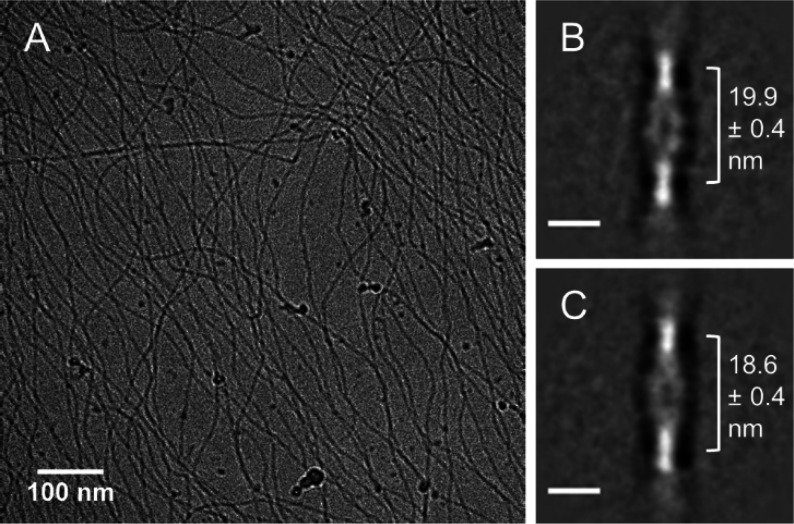
(A) CryoTEM image of **BTA-(S)-D-C**_**12**_**-EG**_**4**_ in water after 1 day of equilibration (*c* = 500 μM). Dark spherical objects originate from ice contamination. (B, C) Class sum image of aligned image extracts of a sample of **BTA-(S)-D-C**_**12**_**-EG**_**4**_ in water after (B) 1 day of equilibration or (C) 4 weeks of equilibration (*c* = 500 μM). The scale bar is 10 nm.

Finally, hydrogen/deuterium exchange followed by mass spectrometry (HDX-MS) experiments were performed to study the physical movement of the monomers between polymers, which we refer to as exchange dynamics.^37,49^ In this technique, aqueous samples of self-assembled BTAs were diluted into D_2_O to study the exchange of labile hydrogen atoms. The three peripheral hydroxyl hydrogen atoms are exchanged immediately for deuterium atoms, whereas the exchange of the amide hydrogen atoms is delayed since they are protected from contact with the solvent by a hydrophobic pocket. The H/D exchange of those amide hydrogen atoms occurs when the monomers are exposed to the aqueous medium, for example by moving between polymers and is therefore a good measure for the exchange of monomers between supramolecular polymers.

An aqueous sample of **BTA-(S)-D-C**_**12**_**-EG**_**4**_ was equilibrated for 1 day in water before a 100× dilution into D_2_O. The H/D exchange of the amides was found to be a slow process for part of the molecules (Figure 3). The initial exchange occurs fast and only a small percentage of BTA4D and BTA5D, which are BTAs with only 1 or 2 of the amide hydrogen atoms exchanged for deuterium atoms, can be observed in the first hour (Figure S7A). This indicates that there are some less-ordered assemblies that undergo fast H/D exchange via solvent penetration. The overall exchange process slows down when 43% of the molecules is deuterated, and after 72 h in D_2_O, 70.0% of the BTAs have all their labile hydrogen atoms exchanged. This is all comparable to the degree and rate of H/D exchange of a sample of **BTA-C**_**12**_**-EG**_**4**_ after 1 day (Figure S8).

**Figure 3:**
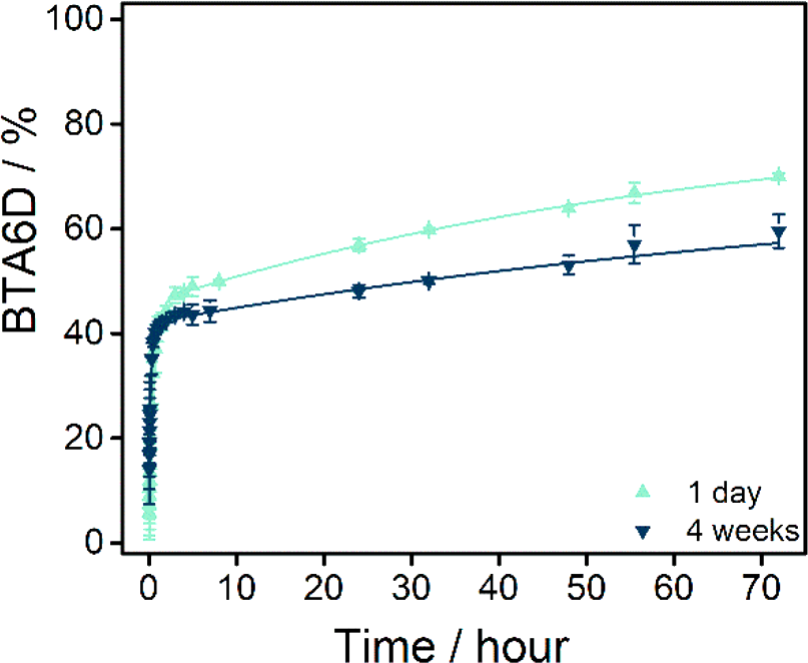
Percentage of fully deuterated **BTA-(S)-D-C**_**12**_**-EG**_**4**_ as a function of time after the 100× dilution of aqueous 500 μM samples into D_2_O (*T* = room temperature). Samples were diluted after 1 day or 4 weeks of equilibration in H_2_O at room temperature. The symbols represent the average and the error bars the standard deviation calculated from three independent measurements. The lines represent a biexponential growth function added to guide the eye.

The HDX-MS experiments were repeated with a sample that was equilibrated for 4 weeks in water before dilution into D_2_O. The H/D exchange of the 4-weeks equilibrated sample follows a similar trend as the 1-day equilibrated sample, with an initial fast exchange and a slow exchange after a few hours (Figure S7B). However, the percentage of BTA4D and BTA5D is lower in this sample, indicating that there is less solvent penetration after equilibration of the sample. Additionally, after 72 h, the percentage of fully deuterated BTAs is only 59.5% compared to 70.0% for the 1-day equilibrated sample (Figure 3). Especially the last part of the H/D exchange is slower after aging, which indicates that the double helices become less dynamic over time. Such a difference in the percentage of BTA6D is not found for a 4-week equilibrated sample of **BTA-C**_**12**_**-EG**_**4**_ (Figure S8).

Taking all measurements together, we conclude that the introduction of isotopic chirality in **BTA-(S)-D-C**_**12**_**-EG**_**4**_ initially does not influence the structure and dynamics of the supramolecular polymers. However, over time the helical bias in the assemblies increases which results in a tighter packing of molecules, a higher degree of order, and as a result less solvent penetration and a slower exchange of monomers. These experiments demonstrate that supramolecular polymers, previously assumed to be in thermodynamic equilibrium, undergo small changes in their molecular packing over several weeks to reach an even more stable state. Many reasons can be proposed for the difference between the achiral and chiral BTA. We propose that the chiral **BTA-(S)-D-C**_**12**_**-EG**_**4**_ prefers to assemble in either P or M helices due to the zero-point energy difference between C–H and C–D stretch vibration next to an amide. Initially, the process does not allow the polymer to reach thermodynamic equilibrium and the maximal helical bias. The latter then increases over time, but this is a slow process at room temperature, probably due to the strong hydrophobic interactions that hold the double helix structure together.^50^ Such a stabilization of the supramolecular polymers was not observed for the achiral **BTA-C**_**12**_**-EG**_**4**_, which lacks the preference for one of the two diastereomerically related helical conformations. The introduction of isotopic chirality is a well-known strategy to obtain information about supramolecular self-assembly in organic solvents,^43,44^ but it is, remarkably, rarely used to study supramolecular polymerization in water. With this work, we would like to motivate others to explore this approach to strengthen our knowledge about supramolecular self-assembly in water. The new insights into the molecular packing, structure, and dynamics of the supramolecular polymers in water stresses the importance of kinetic traps and thermodynamically stable structures.

## Supporting Information Available

Experimental details on synthesis, stereoselectivity and self-assembly, spectroscopy experiments, additional cryoTEM images, details of the HDX-MS experiments, and mixing experiments (PDF)
