# Supplementary material for: Structure and Dynamics of Supramolecular Polymers: Wait and See
Source: ACS Macro Lett. 2022 May 9;11(5):711–5. doi: 10.1021/acsmacrolett.2c00223 (PMC9118549; doi:10.1021/acsmacrolett.2c00223)
Supplement: Supplementary file 1 — mz2c00223_si_001.pdf [file mz2c00223_si_001.pdf]

Supporting Information for:

Structure and Dynamics of Supramolecular Polymers: Wait and See

Sandra M.C. Schoenmakers,[a] A.J.H. Spiering,[a] Svenja Herziger,[b,c] Christoph Böttcher,[c] Rainer Haag,[b] Anja R.A. Palmans[a] and E.W. Meijer*[a]*

[a] Laboratory of Macromolecular and Organic Chemistry, Institute for Complex Molecular Systems, Eindhoven University of Technology, Eindhoven, The Netherlands. Email: a.palmans@tue.nl, e.w.meijer@tue.nl

[b] Institute of Chemistry and Biochemistry, Freie Universität Berlin, Berlin, Germany.

[c] Research Center of Electron Microscopy and Core Facility BioSupraMol, Institute of Chemistry and Biochemistry, Freie Universität Berlin, Berlin, Germany.

Table of Contents:

1. Materials and instrumentation 2
2. Synthesis of BTA-(S)-D-C_{12}-EG_4 5
3. Assessing the enantioselective introduction of deuterium atoms 10
4. Sample preparation for supramolecular polymers in water 11
5. Supporting Figures 12
6. Analysis of the HDX-MS measurements 17
7. References 18

1. Materials and instrumentation

All solvents and chemicals used were of reagent grade quality or better and purchased from Biosolve or Sigma-Aldrich at the highest purity available and used without further purification unless otherwise noted. Reactions were followed by thin-layer chromatography (TLC) using 60-F_{254} silica gel plates from Merck and visualized by UV light at 254 nm and/or staining (ninhydrin, bromocresol green, potassium permanganate, iodine chamber). Water for aqueous samples was purified on an EMD Millipore Milli-Q Integral Water Purification System. NADPH and ADH-T ((S)-selective, 331 U/mL) was purchased from Julich Chiral Solutions GmbH (a Codexis company).

Nuclear Magnetic Resonance (NMR) spectra were recorded on Bruker Ultrashield 400 MHz spectrometers (400 MHz for ^{1}H NMR; 100 MHz for ^{13}C NMR). Deuterated solvents used are indicated in each case. Proton chemical shifts are reported in ppm (δ) downfield from trimethyl silane (TMS) using the resonance frequency of the deuterated solvent ($CDCl_3$ 7.26 ppm) as the internal standard. Peak multiplicities are abbreviated as s: singlet; d: doublet; m: multiplet; t: triplet. Carbon chemical shifts are reported in ppm (δ) downfield from TMS using the resonance frequency of the deuterated solvents ($CDCl_3$ 77.16 ± 0.06 ppm) as the internal standard.

Liquid chromatography mass spectroscopy (LC-MS) spectra were acquired using a device consisting of multiple components: Shimadzu SCL-10 A VP system controller with Shimadzu LC-10AD VP liquid chromatography pumps (with an Alltima C18 3 u (50 x 2.1 mm) reversed-phase column and gradients of water, a Shimadzu DGU 20A3 prominence degasser, a Thermo Finnigan surveyor auto sampler, a Thermo Finnigan surveyor PDA detector and a Thermo Scientific LCW Fleet. All samples were dissolved in 1:1 H_2O:ACN in ca. 0.1 mg/mL concentration.

Matrix assisted laser absorption/ionization mass time of flight mass spectroscopy (MALDI-TOF-MS) spectra were obtained on a Bruker Autoflex Speed. α-Cyano-4-hydroxycinnamic acid (CHCA) or trans-2-[3-(4-tert-butylphenyl)-2-methyl-2-propenylidene]malononitrile (DCBT) were used as matrices. All samples were dissolved in either chloroform or methanol with a concentration of 1.0 mg/mL.

Reversed phase column chromatography was achieved using a reversed phase KP-C_{18}-HS SNAP column on a Biotage Isolera One automated column machine.

Ultraviolet (UV) absorbance spectra were recorded on a Jasco V-650 UV-Vis spectrometer equipped with a Jasco ETCT-762 temperature controller or on a Jasco V-750 UV-Vis spectrometer equipped with a PAC-743 multi-cuvette holder and Jasco ETCT-762 temperature controller. Measurements were performed using Quartz cuvettes (Hellma) with a pathlength of 1 mm (500 μM samples) or 1 cm (50 μM samples). First, a baseline of the corresponding solvent was measured. All measurements were performed with a bandwidth of 1.0 nm, a scan speed of 100

nm/min and a data interval of 0.1 nm, spanning the UV-Vis range from 350 nm to 190 nm. All spectra were averaged over three measurements.

Circular dichroism (CD) and linear dichroism (LD) spectra were measured on a JASCO J-815 CD spectrometer with either a JASCO Peltier MPTC-490S temperature controller with a range of 278 – 373 K or a JASCO Peltier PFD-425S/15 with a range of 263 – 383 K. Measurements were done in Quartz cuvettes (Hellma) with a pathlength of 1 mm (500 μM) or 1 cm (50 μM). A standard sensitivity, a D.I.T of 0.5 seconds, a bandwidth of 1.0 nm, a data pitch of 0.2 nm and a scanning speed of 100 nm/min were used. The spectra were recorded from 350 nm to 190 nm. A baseline of the corresponding solvent was recorded before the measurement.

Fourier-Transform infrared (FT-IR) spectra were recorded on a Perkin Elmer Spectrum Two FT-IR spectrometer. Solid state samples were measured at room temperature from 4000 cm^{-1} to 450 cm^{-1} over 16 scans. Liquid FT-IR measurements were performed using a CaF_2 Liquid Cell with an optical path length of 0.05 mm. Samples for FT-IR in solution were prepared at a concentration of 20 mg/mL to facilitate dissolution. BTA material was weighed and added to a clean vial. All samples were dried overnight with approximately 5 grams of P_2O5 in a separate beaker in the vacuum oven at 40 °C. Sample in MeOD were prepared by adding MeOD to the vials to obtain the desired concentration. The sample in D_2O was prepared by addition of the solvent at the desired concentration, followed by stirring at 80 °C for 15 minutes. The hot and hazy samples were subsequently vortexed for 15 seconds and this procedure was repeated again if the sample still looked hazy. All samples were left to equilibrate at room temperature overnight and samples in D_2O were viscous the next day. First a background of the appropriate solvent was measured. All spectra were measured at room temperature from 400 cm^{-1} to 4000 cm^{-1}, averaged over 64 scans.

Cryogenic transmission electron microscopy (cryoTEM) images were made of vitrified samples with a concentration of 500 μM. Vitrified films were prepared in a 'Vitrobot' instrument (FEI Vitrobot^{TM} Mark IV, FEI Company) at 22 °C and at a relative humidity of 100%. In the preparation chamber of the 'Vitrobot', 3 μL samples were applied on Quantifoil grids (R 2/2, Quantifoil Micro Tools GmbH), which were surface plasma treated just prior to use (Cressington 208 carbon coater operating at 5 mA for 40 s). Excess sample was removed by blotting using filter paper for 3 s with a blotting force of -1, and the thin film thus formed was plunged (acceleration about 3 g) into liquid ethane just above its freezing point. Vitrified films were transferred into the vacuum of a CryoTITAN equipped with a field emission gun that was operated at 300 kV, a post-column Gatan energy filter, and a 2048 x 2048 Gatan CCD camera. Virtrified films were observed in the CryoTITAN microscope at temperatures below -170 °C. Micrographs were taken at low dose conditions, starting at a magnification of 6500 with a defocus setting of -40 μm or at a magnification of 24000 with a defocus setting of -10 μm.

2D image classification and averaging was performed in Berlin on samples with a concentration of 500 µM. The method is based on previously reported procedures.[1] Vitrified films were prepared in a ‘Vitrobot’ instrument (Vitrobot™ Mark IV, Thermo Fisher Scientific) at 22 °C and at a relative humidity of 100%. In the preparation chamber of the ‘Vitrobot’, 4 µL samples were applied on Quantifoil grids (R 1/4) which were surface plasma treated with a BALTEC MED 020. Excess sample was removed by blotting using filter paper for 3.5 s, a drain time of 1.0 s, a wait time of 1.0 s and with a blotting force of -13. The thin film thus formed was plunged (acceleration about 3 g) into liquid ethane just above its freezing point. Vitrified films were imaged with a Talos Arctica™ TEM (Thermo Fisher Scientific) at 200kV accelerating voltage at temperatures below -170 °C. Image recording was done using a Falcon3EC direct electron detector (Thermo Fisher Scientific). Micrographs were taken at low dose conditions, starting at a magnification of 28000x with a defocus setting around -5 µm. From cryo-TEM images, individual motifs (128 x 128 pixels, 0.373 nm/pixel) were extracted using the EMAN tool *boxer*.[2] Utilizing the image processing software package IMAGIC-5,[3] 79 images were aligned with respect to one or multiple reference images, using cross-correlation techniques. The images were furthermore band-pass filtered to exclude low and high spatial frequencies, thus reducing unspecific noise. Subsequently, a mask image was generated, isolating the area of interest in the images. This mask was applied to all images and the multivariate statistical analysis was computed to confirm the absence of other morphologies or artefacts.

Hydrogen deuterium exchange experiments with electrospray ionization were carried out using a Xevo™ G2 QTof mass spectrometer (Waters) with a capillary voltage of 2.7 kV, a cone voltage of 80 V and an extraction cone voltage of 4.0 V. The source temperature was set at 100 °C, the desolvation temperature at 400 °C, and the cone gas flow at 10 L/h and the desolvation gas flow at 500 L/h. The sample solutions subjected to H/D exchange were introduced into the mass spectrometer using a Harvard syringe pump (11 Plus, Harvard Apparatus) at a flow rate of 50 µL/min. The signal was left to equilibrate for 1 measurement before starting the measurement and each measurement was averaged over 1 minute to account for instabilities in the signal. Spectra were recorded in centroid mode and the intensity of the peaks is used for the calculations as described in section 6. Before each measurement, the system was calibrated with a 0.05% H_3PO_4 solution in 1:1 H_2O:ACN. Isotope patterns for calculation were determined with IsoPro software.

2. Synthesis of BTA-(S)-D-C_{12}-EG_4

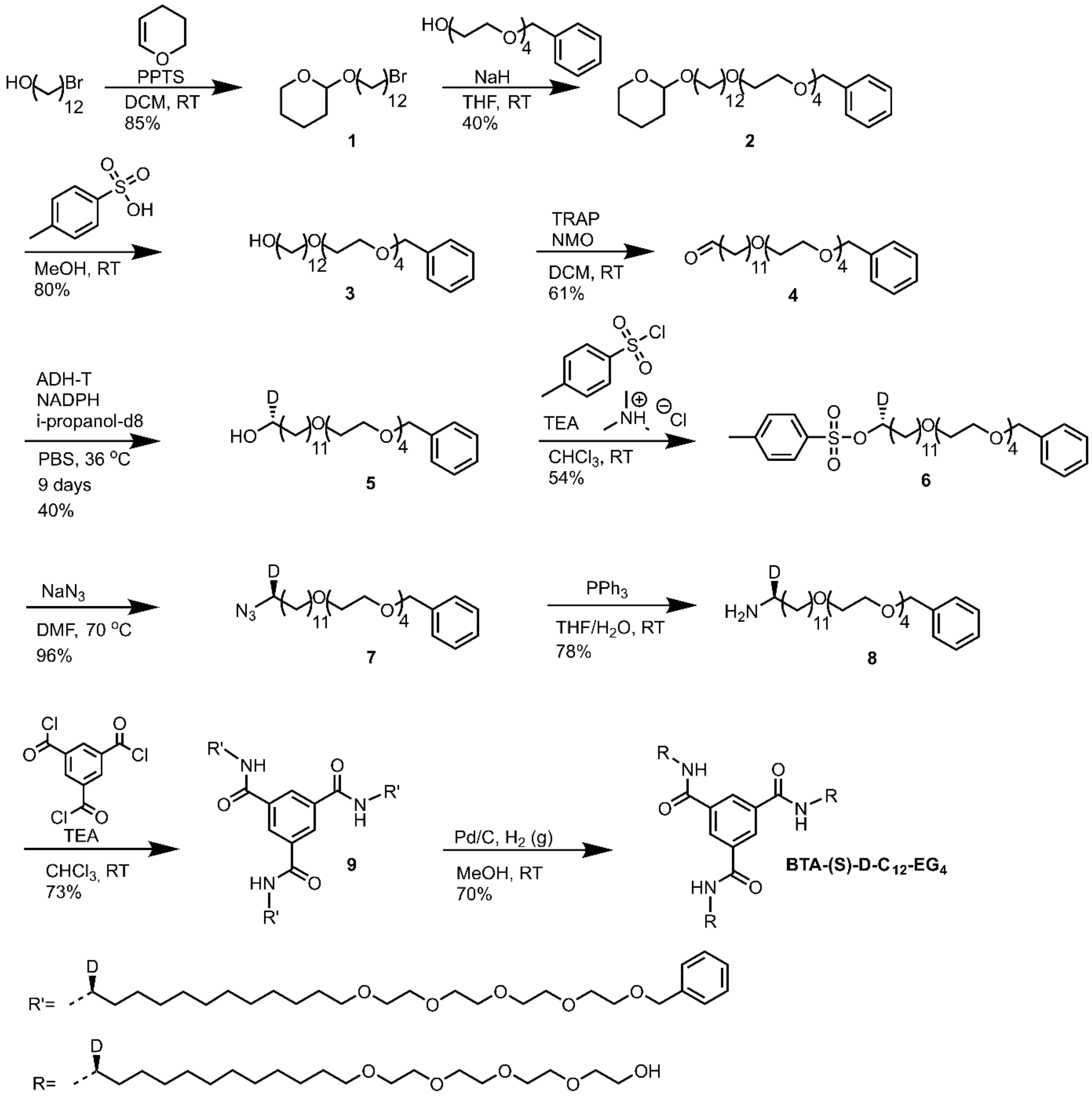

Scheme S1: Synthetic pathway for the synthesis of **BTA-(S)-D-C_{12}-EG_4**.

BTA-(S)-D-C_{12}-EG_4 was synthesized based on previously reported literature procedures[4] (see Scheme S1). A new route towards aldehyde **4** was used to prevent the formation of side products. A deuterium isotope was stereoselectively introduced at the α-position of each dodecyl chain with the alcohol dehydrogenase of *Thermoanaerobacter sp.* (ADH-T, (S)-selective for reduction of deuterated aldehydes with isopropanol, 331 U/mL) with nicotinamide adenine dinucleotide phosphate (NADPH) as a cofactor and isopropanol-d_8 as deuterium source.[5] The formed chiral (*R*)-alcohol is subsequently converted to an (*S*)-amine and coupled to the BTA core.

2-((12-Bromododecyl)oxy)tetrahydro-2H-pyran (1)

A 250 mL round bottom flask was charged with 12-bromo-1-dodecanol (40 mmol, 10.6 g) and DCM (100 mL). 3,4-Dihydropyran (48 mmol, 4.4 mL) and pyridinium *p*-toluenesulfonate (PPTS, 4 mmol, 1.0 gram) were added. The reaction mixture was stirred overnight under argon atmosphere. The reaction mixture was subsequently washed with Na_2CO_3 (aq, 3 x 75 mL) and brine (1 x 75 mL). The solution was dried with $MgSO_4$, filtered and the solvent was evaporated. The crude was purified by column chromatography (500 g SiO_2, heptane/ethyl acetate 95/5 v/v), yielding **1** (11.8 g, 85%). ^{1}H NMR (400 MHz, $CDCl_3$ δ): 4.57 (t, 1H, OC$\underline{H}$O), 3.92-3.82 (m, 1H, DHP OC$\underline{H_2}$CH$_2$), 3.77-3.67 (m, 1H, CH$_2$C$\underline{H_2}$O), 3.54-3.45 (m, 1H, DHP OC$\underline{H_2}$CH$_2$), 3.44-3.34 (m, 3H, CH$_2$C$\underline{H_2}$O, BrC$\underline{H_2}$CH$_2$), 1.92-1.77 (m, 3H, DHP OCHC$\underline{H_2}$, BrCH$_2$C$\underline{H_2}$), 1.77-1.65 (m, 1H, OCHC$\underline{H_2}$), 1.63-1.18 (m, 22H, DHP OCH$_2$C$\underline{H_2}$C$\underline{H_2}$, aliphatic). ^{13}C NMR (100 MHz, $CDCl_3$ δ): 98.86, 67.70, 62.36, 34.05, 32.86, 30.81, 29.77, 29.55, 29.52, 29.48, 29.43, 28.77, 28.19, 26.25, 25.33, 19.73. FT-IR (ATR) *v* (cm-1): 2923, 2853, 1465, 1440, 1352, 1322, 1259, 1200, 1184, 1152, 1135, 1120, 1078, 1032, 1022, 985, 905, 869, 815, 722, 646, 563.

2-((1-Phenyl-2,5,8,11,14-pentaoxahexacosan-26-yl)oxy)tetrahydro-2H-pyran (2)

A 500 mL round bottom flask was charged with monobenzyl ether tetraethylene glycol (31.5 mmol, 8.9 g) and THF (100 mL) and the flask was cooled in an ice bath. NaH (34 mmol, 1.37 g) was added in portions and the reaction mixture was stirred at room temperature for 1 hour. The mixture was cooled again with an ice bath and **1** (28.6 mmol, 10 g) in THF (50 mL) was added dropwise. The reaction mixture was stirred overnight at room temperature. Water (300 mL) was added and the mixture was extracted with ethyl acetate (3x100 mL). The organic layer was washed with water (100 mL), dried with $MgSO_4$, filtered and the solvent was evaporated. The crude was purified by column chromatography (550 g SiO_2, heptane/ethyl acetate 50/50 v/v), yielding **2** (6.3 g, 40%). ^{1}H NMR (400 MHz, $CDCl_3$ δ): 7.37-7.27 (m, 5H, Ar), 4.57 (m, 3H, ArC$\underline{H_2}$O, DHP OC$\underline{H}$O), 3.92-3.81 (m, 1H, DHP OC$\underline{H_2}$CH$_2$), 3.80-3.30 (m, 21H, DHP OC$\underline{H_2}$CH$_2$, CH$_2$C$\underline{H_2}$O, O(C$\underline{H_2}$)$_2$O, CH$_2$OC$\underline{H_2}$), 1.90-1.64 (m, 2H, DHP CHC$\underline{H_2}$), 1.63-1.46 (m, 8H, DHP OCH$_2$C$\underline{H_2}$C$\underline{H_2}$, OCH$_2$C$\underline{H_2}$), 1.42-1.19 (m, 16H, aliphatic). ^{13}C NMR (100 MHz, $CDCl_3$ δ): 138.30, 128.35, 127.73, 127.57, 98.64, 73.25, 71.56, 70.67, 70.64, 70.06, 69.46, 67.70, 62.34, 30.81, 29.77, 29.66, 29.61, 26.51, 26.26, 26.11, 25.53, 19.72. FT-IR (ATR) *v* (cm-1): 2924, 2854, 1496, 1454, 1351, 1323, 1285, 1252, 1201, 1183, 1111, 1078, 1030, 988, 905, 869, 815, 735, 698, 614, 542, 466. MALDI-TOF-MS: *m/z* calculated for $C_{32}H_{56}O_7+Na^+$: 575.39 [M+Na]$^+$; observed 575.40.

1-Phenyl-2,5,8,11,14-pentaoxahexacosan-26-ol (3)

A 500 mL round bottom flask was charged with **2** (5.4 mmol, 3 g), *p*-toluenesulfonic acid monohydrate (1.2 mmol, 230 mg) and MeOH (20 mL). The reaction mixture was stirred overnight at room temperature. Water (20 mL) and $NaHCO_3$ (s) was added until basic pH was reached. MeOH was evaporated, DCM (100 mL) was added and the mixture was washed with water (75 mL) and brine (75 mL). The mixture was dried with Na_2SO_4, filtered and the solvent was evaporated, yielding **3** as a clear oil (2.4 g, 80%).

^{1}H NMR (400 MHz, $CDCl_3$ δ): 7.39-7.27 (m, 5H, Ar), 4.56 (s, 2H, ArC<u>H</u>$_2$O), 3.72-3.51 (m, 18H, O(C<u>H</u>$_2$)$_2$O, CH_2C<u>H</u>$_2$O), 3.43 (t, 2H, CH_2C<u>H</u>$_2$OH), 1.64-1.49 (m, 4H, C<u>H</u>$_2$$CH_2$OH), C<u>H</u>$_2$$CH_2$O), 1.41-1.19 (m, 16H, aliphatic).

1-Phenyl-2,5,8,11,14-pentaoxahexacosan-26-al (4)
A 250 mL round bottom flask was charged with **3** (7 mmol, 3.28 g), *N*-methylmorpholine *N*-oxide (NMO, 10.5 mmol, 1.23 g), 4 Å molsieves (crushed, 3.5 g) and DCM (14 mL). Tetrapropylammonium perruthenate (TRAP, 0.35 mmol, 123 mg) was slowly added and the reaction mixture was stirred for 1.5 hours. The reaction mixture was filtered over a filtration plug (Celite, SiO_2, Celite) on a glass filter and was thoroughly washed with ethyl acetate. The solvent was evaporated, and the crude was subsequently purified by column chromatography (150 g SiO_2, heptane/ethyl acetate 30/70 v/v), yielding **4** (2.0 g, 61%). ^{1}H NMR (400 MHz, $CDCl_3$ δ): 9.76 (s, 1H, C<u>H</u>O), 7.39-7.27 (m, 5H, Ar), 4.57 (s, 2H, ArC<u>H</u>$_2$O), 3.73-3.52 (m, 16H, O(C<u>H</u>$_2$)$_2$O), 3.48-3.37 (t, 2H, CH_2C<u>H</u>$_2$O), 2.47-2.33 (m, 2H, C<u>H</u>$_2$CHO), 1.69-1.49 (m, 4H, C<u>H</u>$_2$$CH_2$CHO, C<u>H</u>$_2$$CH_2$O), 1.37-1.12 (m, 14H, aliphatic). ^{13}C NMR (100 MHz, $CDCl_3$ δ): 202.93, 138.30, 128.35, 127.73, 127.57, 73.25, 71.54, 70.67, 70.63, 70.07, 69.45, 43.92, 29.65, 29.56, 29.51, 29.48, 29.41, 29.35, 29.17, 26.10, 22.09. FT-IR (ATR) *v* (cm-1): 2924, 2854, 2718, 1724, 1496, 1454, 1410, 1383, 1351, 1328, 1296, 1247, 1205, 1101, 1041, 1028, 993, 946, 847, 851, 736, 698, 610, 523. MALDI-TOF-MS: *m/z* calculated for $C_{27}H_{46}O_6$+Na^+: 489.32 $[M+Na]^+$; observed 489.37.

(*R*)-1-Phenyl-2,5,8,11,14-pentaoxahexacosan-26-*d*-26-ol (5)
A 250 round bottom flask was charged with **4** (4.65 mmol, 2.17 g) and isopropanol-d8 (10 mL). Phosphate buffered saline (PBS, 50 mL) and nicotinamide adenine dinucleotide phosphate (NADP, 0.28 mmol, 208 mg) were added. The turbid mixture was stirred at 36 °C and the enzyme ADH-T (311 U/mL, 0.2 mL) was added. The reaction became clear after 1 hour. The reaction was checked with LCMS after 6 days, which showed aldehyde, alcohol and carboxylic acid derivatives. More NADHP (0.2 mmol, 200 mg) and ADH-T (0.2 mL) were added but the ratio between aldehyde, alcohol and carboxylic acid did not improve after 3 days. The reaction mixture was cooled, extracted with chloroform (3 x 40 mL), dried with $MgSO_4$, filtered and the solvent was evaporated. The crude material was purified by reverse phase column chromatography (2 x 120 g C_{18} column, water/acetonitrile 90/10-20/80 v/v). Fractions were checked with LC-MS and fraction with pure alcohol (100 mg) and with alcohol and some carboxylic acid impurity (775 mg, impurity can be removed after tosylation) were combined and lyophilized. ^{1}H NMR (400 MHz, $CDCl_3$ δ): 7.38-7.27 (m, 5H, Ar), 4.56 (s, 2H, ArC<u>H</u>$_2$O), 3.73-3.52 (m, 17H, O(C<u>H</u>$_2$)$_2$O, C<u>H</u>DOH), 3.48-3.37 (t, 2H, CH_2C<u>H</u>$_2$O), 1.69-1.48 (m, 4H, C<u>H</u>$_2$CHDOH, C<u>H</u>$_2$$CH_2$O), 1.42-1.06 (m, 16H, aliphatic). ^{13}C NMR (100 MHz, $CDCl_3$ δ): 138.28, 128.35, 127.74, 127.58, 73.25, 71.55, 70.66, 70.63, 70.61, 70.06, 69.44, 62.93, 62.71, 62.50, 32.72, 29.64, 29.59, 29.57, 29.48, 29.42, 26.09, 25.72. FT-IR (ATR) *v* (cm-1): 3467 (b), 2924, 2854, 2132, 1722, 1454 1351, 1297, 1249, 1205, 1098, 1043, 1028, 942, 879, 852, 736, 698, 607, 527, 464.

LC-MS: *m/z* calculated for $C_{27}H_{47}DO_6+H^+$: 469.35 $[M+H]^+$; observed 470.33; *m/z* calculated for $C_{27}H_{46}O_7+H^+$: 482.32 $[M+H]^+$; observed 483.25.

(*R*)-1-Phenyl-2,5,8,11,14-pentaoxahexacosan-26-yl-26-*d*-4-methylbenzenesulfonate (6)
A 100 mL round bottom flask was charged with **5** (0.7 mmol, 330 mg), triethylamine (1.4 mmol, 0.2 mL), trimethylamine hydrochloride (0.14 mmol, 8.5 mg). The mixture was cooled with an ice bath and *p*-toluenesulfonylchloride (0.98 mmol, 200 mg) in chloroform (10 mL) was added dropwise. Additional amines and *p*-toluenesulfonylchloride were added after 4 hours to reach completion and the mixture was stirred overnight. More chloroform was added and the mixture was washed with water (3x) and brine (1x). The solution was dried with $MgSO_4$, filtered and the solvent was evaporated. The crude was purified with column chromatography (25 g SiO_2, heptane/ethyl acetate 40/60 v/v), yielding **6** as a slightly yellow oil (310 mg, 54%). ^1H NMR (400 MHz, $CDCl_3$ δ): 7.78 (d, 2H, Ar tosyl), 7.38-7.27 (m, 7H, Ar tosyl, benzyl), 4.56 (s, 2H, $ArC\underline{H_2}O$), 3.99 (t, 1H, CHD), 3.73-3.50 (m, 16H, $O(C\underline{H_2})_2O$), 3.47-3.37 (t, 2H, $CH_2C\underline{H_2}O$), 2.44 (s, 3H, $C\underline{H_3}$) 1.70-1.49 (m, 4H, $C\underline{H_2}CHD$, $C\underline{H_2}CH_2O$), 1.35-1.10 (m, 16H, aliphatic). ^{13}C NMR (100 MHz, $CDCl_3$ δ): 144.58, 138.30, 133.29, 129.79, 128.35, 127.88, 127.73, 127.58, 73.25, 71.54, 70.67, 70.63, 70.39, 70.16, 70.06, 69.45, 29.66, 29.58, 29.54, 29.49, 29.39, 28.94, 28.72, 26.10, 25.31, 21.65. MALDI-TOF-MS: *m/z* calculated for $C_{34}H_{53}DO_8S+Na^+$: 646.35 $[M+Na]^+$; observed 646.38;

(*S*)-26-Azido-1-phenyl-2,5,8,11,14-pentaoxahexacosane-26-*d* (7)
A 25 mL round bottom flask was charged with **6** (0.47 mmol, 293 mg) in DMF (5 mL). Sodium azide (1.49 mmol, 96 mg) was added and the reaction mixture was stirred overnight at 70 °C under argon atmosphere. Brine (20 mL) was added to the mixture and it was extracted with chloroform (2 x 10 mL). The organic layers were washed with water and brine, dried with $MgSO_4$, filtered and the solvent was evaporated, yielding **7** as an orange oil (223 mg, 96%). ^1H NMR (400 MHz, $CDCl_3$ δ): 7.38-7.27 (m, 5H, Ar), 4.57 (s, 2H, $ArC\underline{H_2}O$), 3.73-3.52 (m, 16H, $O(C\underline{H_2})_2O$), 3.43 (t, 2H, $CH_2C\underline{H_2}O$), 2.42 (t, 1H, $C\underline{H}D$), 1.65-1.50 (m, 4H, $C\underline{H_2}CHD$, $C\underline{H_2}CH_2O$), 1.43-1.10 (m, 16H, aliphatic). ^{13}C NMR (100 MHz, $CDCl_3$ δ): 138.32, 128.35, 127.73, 127.57, 73.26, 71.55, 70.68, 70.65, 70.64, 70.08, 69.48, 51.39, 51.17, 50.96, 29.66, 29.58, 29.56, 29.52, 29.49, 29.47, 29.16, 28.74, 26.70, 26.11. FT-IR (ATR) *v* (cm-1): 2925, 2855, 2092, 1723, 1454, 1351, 1254, 1102, 1028, 946, 880, 850, 736, 698, 616, 557, 464. MALDI-TOF-MS: *m/z* calculated for $C_{27}H_{46}DN_3O_5+Na^+$: 517.35 $[M+Na]^+$; observed 517.37.

(*S*)-1-Phenyl-2,5,8,11,14-pentaoxahexacosan-26-*d*-26-amine (8)
A 25 mL round bottom flask was charged with **7** (0.42 mmol, 210 mg), THF (3.5 mL) and water (0.1 mL). Triphenylphosphine (1.3 mmol, 350 mg) was added and the reaction was stirred overnight with a $CaCl_2$ tube. The solvent was evaporated and the crude was purified by column chromatography (SiO_2, chloroform/methanol/2-propylamine 100/0/0 –97.5/2.5/0 – 85/5/10 v/v/v), yielding **8** (156 mg, 78%). ^1H NMR (400 MHz, $CDCl_3$ δ): 7.38-7.27 (m, 5H, Ar), 4.57 (s, 2H, $ArC\underline{H_2}O$), 3.73-3.53 (m, 16H,

O(C$\underline{H}_2$)$_2$O), 3.43 (t, 2H, $CH_2C\underline{H}_2O$), 2.71 (t, 1H, C$\underline{H}$D), 1.63-1.53 (m, 2H, $C\underline{H}_2CH_2O$), 1.53-1.43 (m, 2H, $C\underline{H}_2CHD$), 1.38-1.14 (m, 16H, aliphatic). ^{13}C NMR (100 MHz, $CDCl_3$ δ): 138.24, 128.36, 127.77, 127.60, 73.25, 71.54, 70.64, 70.63, 70.60, 70.58, 70.04, 69.43, 65.85, 41.51, 41.31, 41.10, 32.15, 29.61, 29.51, 29.49, 29.42, 29.33, 26.75, 26.05, 15.28. LC-MS: *m/z* calculated for $C_{27}H_{48}DNO_5$+Na^+: 469.38 $[M+Na]^+$; observed 469.50.

N^1,N^3,N^5-Tris((*S*)-1-phenyl-2,5,8,11,14-pentaoxahexacosan-26-yl-26-*d*)benzene-1,3,5-tricarboxamide (9)

A 25 mL round bottom flask was charged with **8** (0.31 mmol, 145 mg) and chloroform (2.5 mL). Triethylamine (0.37 mmol, 0.052 mL) was added. The reaction mixture was cooled with an ice bath and 1,3,5-benzene tricarbonyl trichloride (0.09 mmol, 24 mg) in chloroform (0.5 mL) was added dropwise. The reaction was stirred overnight at room temperature. Additional chloroform was added and the mixture was washed with HCl (1M, 2x) and brine (1x). The solution was dried with $MgSO_4$, filtered and the solvent was evaporated. The crude material was purified by column chromatography (9 g SiO_2, dichloromethane/methanol 97/3 v/v). A small impurity was observed so 2 additional columns were performed (4.8 g SiO_2, heptane/ethylene glycol dimethyl ether (distillated), 50/50 v/v), yielding **9** (102 mg, 73%). ^{1}H NMR (400 MHz, $CDCl_3$ δ): 8.38 (s, 3H, BTA Ar), 7.38-7.27 (m, 15H, benzyl Ar), 6.66 (s, 3H, N$\underline{H}$), 4.54 (s, 6H, $ArC\underline{H}_2O$), 3.72-3.49 (m, 48H, O(C$\underline{H}_2$)$_2$O), 3.51-3.33 (m, 9H, $CH_2C\underline{H}_2O$, C$\underline{H}$D), 1.67-1.45 (m, 12H, $C\underline{H}_2CH_2O$, $C\underline{H}_2CHD$), 1.41-1.17 (m, 48H, aliphatic). ^{13}C NMR (100 MHz, $CDCl_3$ δ): 165.71, 138.20, 135.26, 129.68, 128.34, 128.08, 127.75, 127.59, 73.25, 71.54, 70.66, 70.64, 70.61, 70.05, 69.43, 29.60, 29.52, 29.47, 29.45, 29.42, 29.25, 26.94, 26.06. MALDI-TOF-MS: *m/z* calculated for $C_{90}H_{144}D_3N_3O_{18}$+$Na^+$: 1584.08 $[M+Na]^+$; observed 1584.11.

BTA-(S)-D-C_{12}-EG_4

A 25 mL round bottom flask was charged with **9** (0.066 mmol, 104 mg) and methanol (4 mL). N_2 (g) was bubbled through the solution and Pd/C (10 wt%, 12 mg) was added. The reaction mixture was stirred under H_2 (g) atmosphere overnight. The mixture was subsequently filtered over a glass filter with Celite and thoroughly washed with MeOH. The solvent was removed and the crude material was purified by reverse phase column chromatography (12 g C_{18}, water/acetonitrile 90/10-20/80 v/v). The pure fractions were lyophilized to yield **BTA-(S)-D-C_{12}-EG_4** (60 mg, 70%) as a white fluffy powder. ^{1}H NMR (400 MHz, $CDCl_3$ δ): 8.37 (s, 3H, BTA Ar), 6.78 (m, 3H, N$\underline{H}$), 3.78-3.50 (m, 48H, O(C$\underline{H}_2$)$_2$O), 3.49-3.35 (m, 9H, $CH_2C\underline{H}_2O$, C$\underline{H}$D), 2.86 (s, 3H, O$\underline{H}$), 1.65-1.47 (m, 12H, $C\underline{H}_2CH_2O$, $C\underline{H}_2CHD$), 1.44-1.14 (m, 48H, aliphatic). ^{13}C NMR (100 MHz, $CDCl_3$ δ): 165.83, 135.23, 128.12, 72.55, 71.55, 70.61, 70.56, 70.32, 70.02, 61.71, 29.55, 29.50, 29.45, 29.42, 29.39, 29.23, 26.92, 26.03. MALDI-TOF-MS: *m/z* calculated for $C_{69}H_{126}D_3N_3O_{18}$+$Na^+$: 1313.94 $[M+Na]^+$; observed 1313.96.

3. Assessing the enantioselective introduction of deuterium atoms

The stereoselectivity of chiral (*R*)-alcohol **5** was studied with (*R*)-α-methoxy-α-trifluoromethylphenylacetyl chloride (MTPA-Cl) derivatization of the alcohol (Scheme S2).[5] With this tool diastereomeric esters are formed which facilitates the determination of the *ee* of the chiral alcohol.

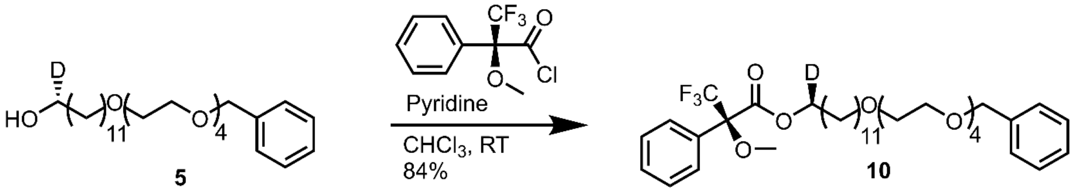

Scheme S2: Synthetic scheme for the MTPA derivation of **5**.

(*S*)-1-Phenyl-2,5,8,11,14-pentaoxahexacosan-26-yl-26-*d*-3,3,3-trifluoro-2-methoxy-2-phenylpropanoate (10)

A 10 mL round bottom flask was charged with **5** (0.09 mmol, 42 mg, no carboxylic acid impurity) and chloroform (0.3 mL). Pyridine (0.3 mL, dry) was added slowly with a syringe. (*R*)-MPTA-Cl (0.1 mmol, 0.02 mL) was added dropwise with a syringe as well and the reaction mixture was stirred under argon atmosphere overnight. Water (5 drops) were added and the mixture was stirred for 15 minutes. It was then diluted with ether (8 mL) and washed with HCL (1M), Na_2SO_3 (sat.) and brine. The organic layer was dried with $MgSO_4$, filtered and the solvent was evaporated, yielding **10** (51 mg, 84%). ^{1}H NMR (400 MHz, $CDCl_3$ δ): 7.57-7.29 (m, 10H, Ar), 4.57 (s, 2H, ArC$\underline{H_2}$O), 4.31 (t, 1H, C$\underline{H}$D), 3.75-3.50 (m, 19H, O(C$\underline{H_2}$)$_2$O, C$\underline{H_3}$), 3.43 (t, 2H, CH_2C$\underline{H_2}$O), 1.74-1.63 (m, 2H, C$\underline{H_2}$CHD), 1.61-1.53 (m, 2H, C$\underline{H_2}$$CH_2$O), 1.39-1.14 (m, 16H, aliphatic). ^{13}C NMR (100 MHz, $CDCl_3$ δ): 166.61, 138.27, 132.40, 129.56, 128.38, 128.35, 127.73, 127.58, 127.31, 124.76, 121.89, 84.72, 84.44, 73.25, 71.55, 70.65, 70.61, 70.05, 69.44, 66.48, 66.25, 66.04, 65.86, 55.43, 45.61, 29.64, 29.58, 29.55, 29.49, 29.42, 29.06, 28.23, 26.10, 25.67, 15.27, 8.51. MALDI-TOF-MS: *m/z* calculated for $C_{37}H_{54}DF_3O_8+Na^+$: 708.38 $[M+Na]^+$; observed 708.39.

The OC-H proton resonates at 4.31 ppm in the ^{1}H NMR spectrum which corresponds to the (*R*)-alcohol as reported in literature.[5] No overlap of the (S)-alcohol was detected, indicating that the deuterium atom was introduced with an enantiomeric excess of > 95%. Based on literature reports we assume that this high stereoselectivity does not decrease upon conversion to the (S)-amine.[5]

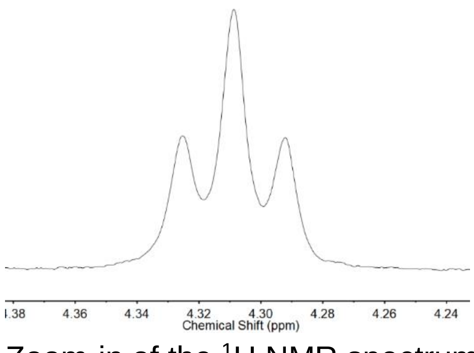

Zoom-in of the ^{1}H NMR spectrum of **10** to visualize the OC-*H* peaks.

4. Sample preparation for supramolecular polymers in water

Sample preparation of BTA-C_{12}-EG_4 and BTA-(S)-D-C_{12}-EG_4: the solid material was weighed into a glass vial equipped with a magnetic stirring bar. MQ-water was added to obtain the desired concentration. The sample was subsequently stirred at 80 °C for 15 minutes and the hot and hazy sample was vortexed immediately afterwards for 15 seconds. All samples were left to equilibrate at room temperature.

Sample preparation of mixtures: 2.5 mM stock solutions of the BTAs were prepared in ACN. Stock solutions were mixed in the desired ratio in a separate vial with magnetic stirring bar. ACN was evaporated with a stream of N_2 (g) in samples. MQ-water was added to obtain the desired concentration. The sample was subsequently stirred at 80 °C for 15 minutes and the hot and hazy sample was vortexed immediately afterwards for 15 seconds. All samples were left to equilibrate at room temperature.

5. Supporting Figures

A **B**

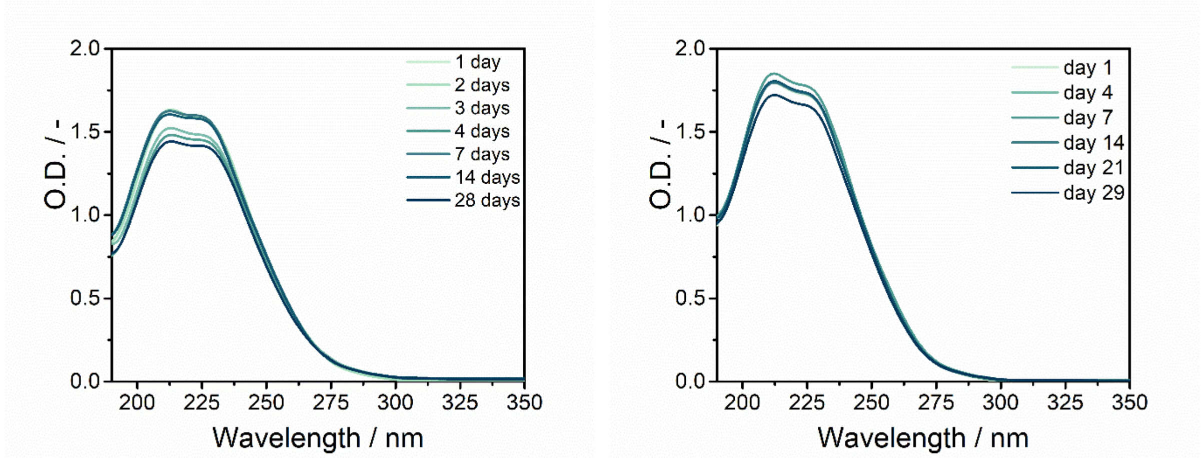

Figure S1: UV spectra of **BTA-(S)-D-C12-EG4** in water over time at (A) 50 µM concentration (l = 1 cm, T = 20 °C) and (B) 500 µM concentration (l = 1 mm, T = 20 °C). The maxima at 211 and 229 nm are observed at both concentrations and is identical to the maxima observed for supramolecular polymers formed by **BTA-C12-EG4**.[6]

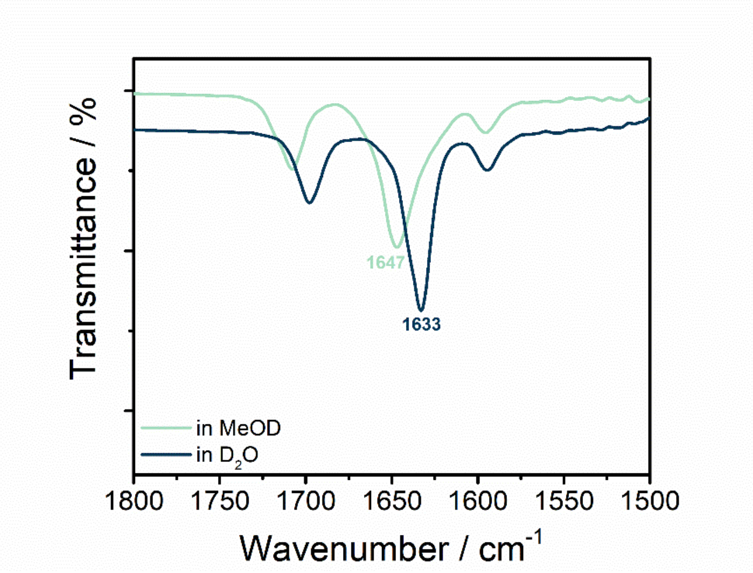

Figure S2: Zoom of the FT-IR spectrum of the amide I vibration of **BTA-(S)-D-C12-EG4** in MeOD and D_2O (c = 20 mg/mL, l = 0.05 mm, T = room temperature). The peak at 1647 cm^{-1} corresponds to the absence of intermolecular hydrogen bonds, whereas the peak at 1633 cm^{-1} corresponds to hydrogen bonded supramolecular polymers, thereby confirming the formation of intermolecular hydrogen bonds in aqueous solution.[7] The vibrations at 1708 cm^{-1} in MeOD[8] and 1697 cm^{-1} in D_2O[9] originate from the C=O vibration of acetone which was used to clean the cuvette.

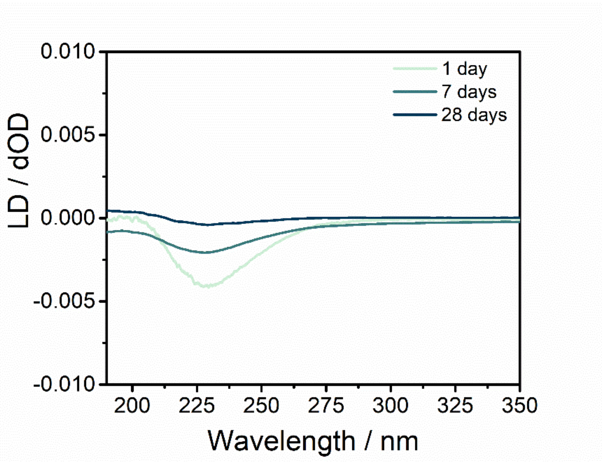

Figure S3: LD spectra of **BTA-(S)-D-C_{12}-EG_4** in water over time (c = 50 µM, l = 1 cm, T = 20 °C).

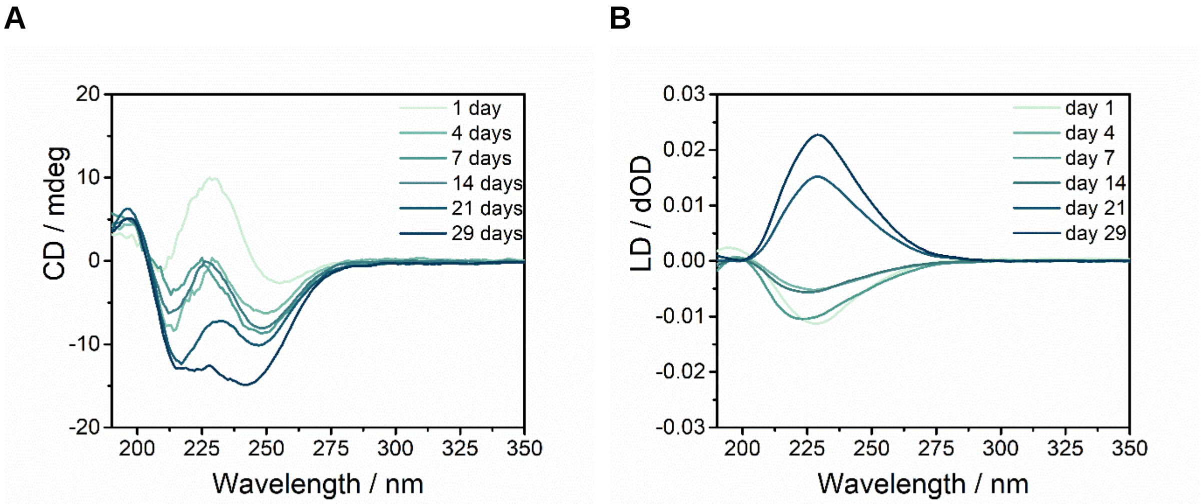

Figure S4: (A) CD and (B) LD spectra of **BTA-(S)-D-C_{12}-EG_4** (c = 500 µM, l = 1 mm, T = 20 °C) in water over time. The increase of the Cotton effects at 213 and 250 nm over time is similar to the increase reported at 50 µM concentration (Figure 1). However, the band at 230 nm is significantly more influenced by LD at higher concentrations as is visualized in this figure.

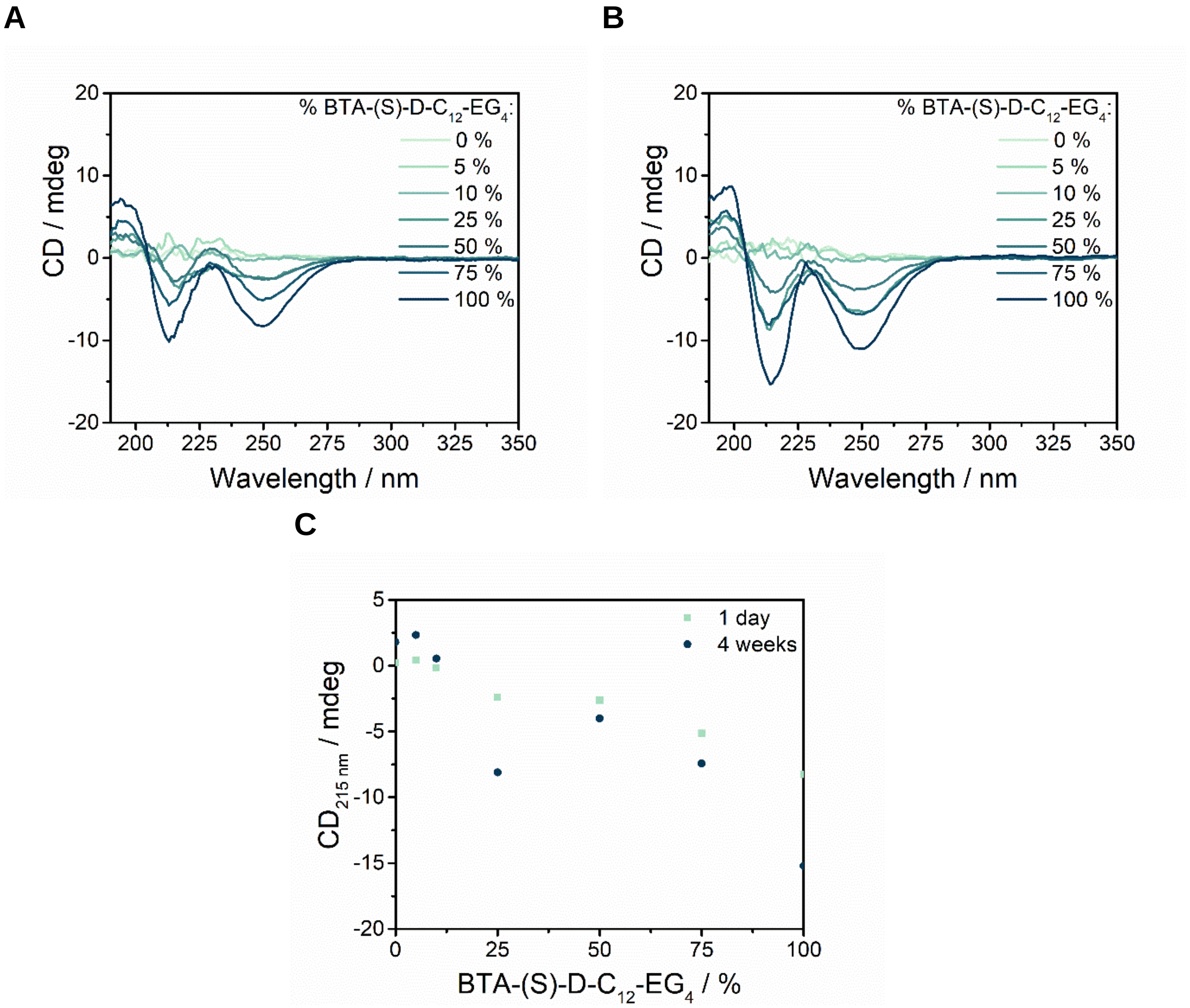

Figure S5: (A) CD spectra of mixtures of **BTA-C12-EG4** with **BTA-(S)-D-C12-EG4** in water after 1 day of equilibration (c_{total} = 50 µM, l = 1 cm, T = 20°C). (B) CD spectra of mixtures of **BTA-C12-EG4** with **BTA-(S)-D-C12-EG4** in water after 4 weeks of equilibration (c_{total} = 50 µM, l = 1 cm, T = 20°C). The sample with 25% of **BTA-(S)-D-C12-EG4** shows a larger CD signal than expected dure to a larger LD signal. (C) CD signal at 215 nm for the mixtures of **BTA-C12-EG4** with **BTA-(S)-D-C12-EG4** in water. The CD signal decreases linearly with the percentage of **BTA-(S)-D-C12-EG4**, indicating that there is no amplification of asymmetry upon addition of the chiral BTA.

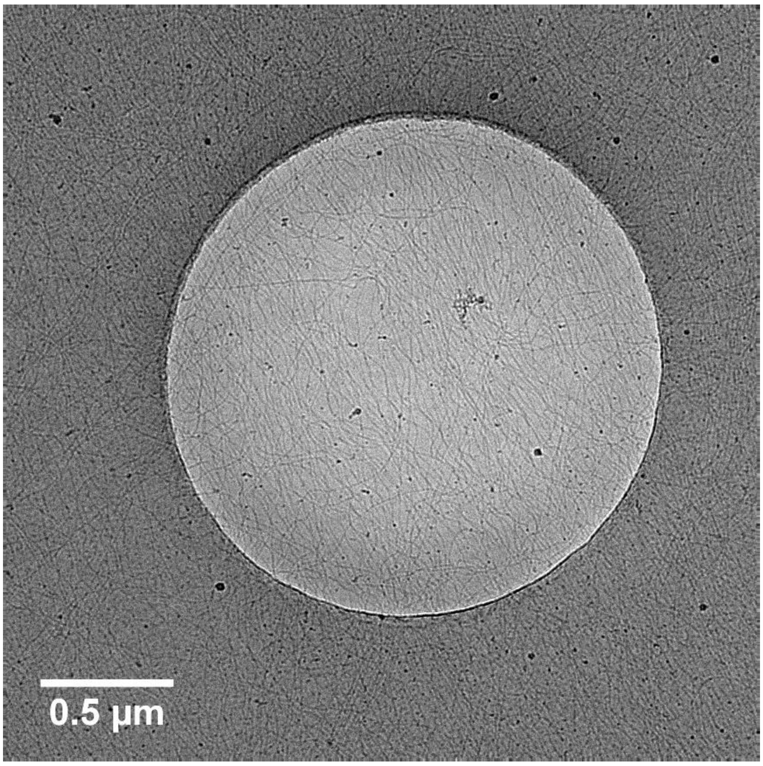

Figure S6: CryoTEM image of **BTA-(S)-D-C_{12}-EG_4** in water after 1 day of equilibration (c = 500 µM). Dark spherical objects originate from ice contamination. The zoomed-out image shows their micrometer length.

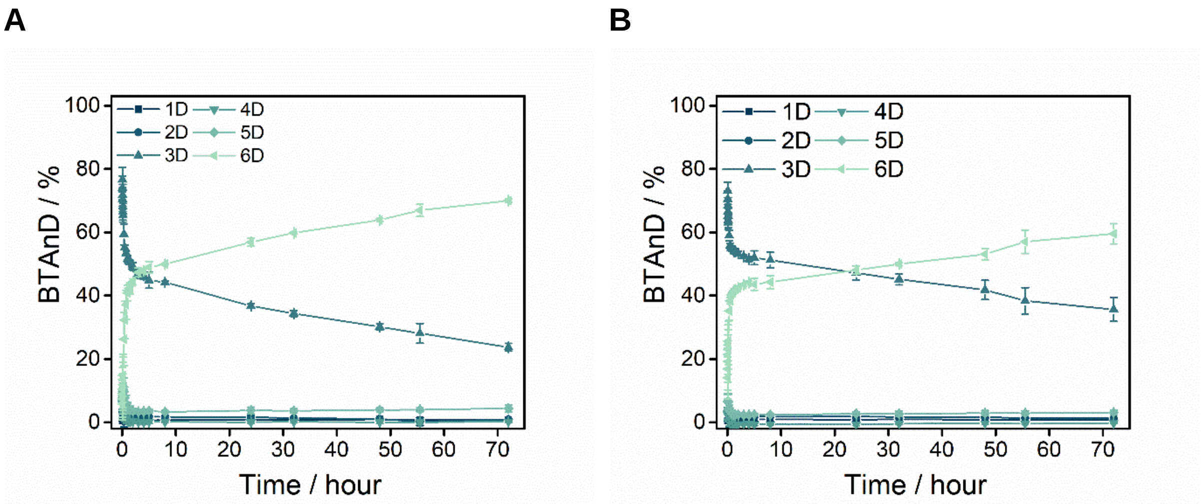

Figure S7: The percentage of different deuterated analogues of **BTA-(S)-D-C_{12}-EG_4** as a function of time after the 100x dilution of an aqueous 500 µM sample into D_2O (T = room temperature). The sample was diluted after (A) 1 day or (B) 4 weeks of equilibration in H_2O. The symbols represent the average and the error bars the standard deviation calculated from three independent measurements. The lines are added to guide the eye.

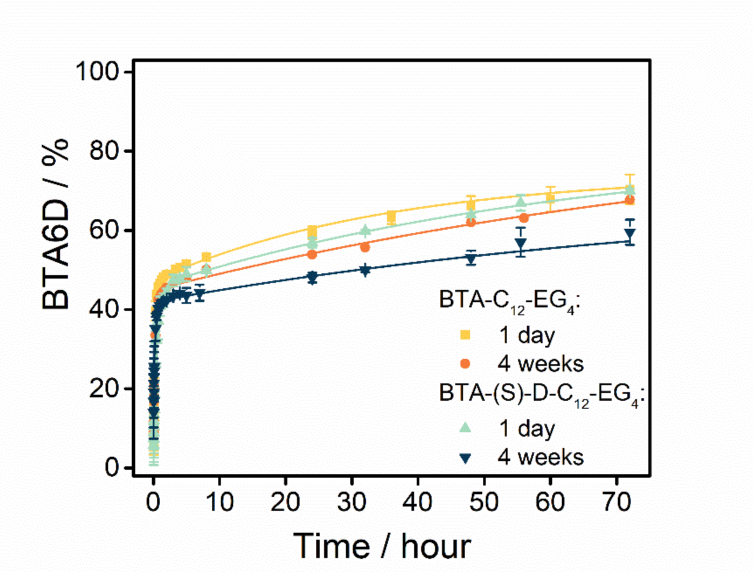

Figure S8: The percentage of fully deuterated **BTA-C12-EG4** and **BTA-(S)-D-C12-EG4** as a function of time after the 100x dilution of aqueous 500 µM samples into D_2O (T = room temperature). Samples were diluted after 1 day or 4 weeks of equilibration in H_2O at room temperature. The symbols represent the average and the error bars the standard deviation calculated from three independent measurements (except for the sample of **BTA-C12-EG4** equilibrated for 4 weeks). The lines represent a bi-exponential growth function added to guide the eye.

6. Analysis of the HDX-MS measurements

All HDX-MS experiments were performed under similar conditions to eliminate influences from the environment, for example temperature. The measurements were performed by diluting a sample of self-assembled supramolecular building-blocks in H_2O 100x into D_2O. 0.5 mM sodium acetate was added to the D_2O prior to the dilution to facilitate the detection. In all cases the distributions with two sodium ions were used for the calculations. All calculations are based on the method presented in previous literature for **BTA-C$_{12}$-EG$_4$**.[10]

Isotope correction for **BTA-(S)-D-C$_{12}$-EG$_4$** is based on the following set of equations:

$I_{BTA1D\_c} = I_{668.97}$

$I_{BTA2D\_c} = I_{669.47} - 0.80I_{BTA1D\_c}$

$I_{BTA3D\_c} = I_{669.97} - 0.80I_{BTA2D\_c} - 0.36I_{BTA1D\_c}$

$I_{BTA4D\_c} = I_{670.48} - 0.80I_{BTA3D\_c} - 0.36I_{BTA2D\_c} - 0.11I_{BTA1D\_c}$

$I_{BTA5D\_c} = I_{670.98} - 0.80I_{BTA4D\_c} - 0.36I_{BTA3D\_c} - 0.11I_{BTA2D\_c} - 0.03I_{BTA1D\_c}$

$I_{BTA6D\_c} = I_{671.48} - 0.80I_{BTA5D\_c} - 0.36I_{BTA4D\_c} - 0.11I_{BTA3D\_c} - 0.03I_{BTA2D\_c} - 0.01I_{BTA1D\_c}$

with $I_{668.97}$, $I_{669.47}$, $I_{669.97}$, $I_{670.48}$, $I_{670.98}$ and $I_{671.48}$ the intensity at m/z = 668.97, 669.47, 669.97, 670.48, 670.98 and 671.48, respectively.

The correction for the presence of 1 vol% H_2O can be calculated with the following set of equations:

$I_{BTA1D} = I_{BTA1D\_c} - 6.31\times10^{-10}I_{BTA6D\_c} - 3.06\times10^{-4}I_{BTA3D\_c}$

$I_{BTA2D} = I_{BTA2D\_c} - 1.56\times10^{-7}I_{BTA6D\_c} - 3.03\times10^{-2}I_{BTA3D\_c}$

$I_{BTA3D} = I_{BTA3D\_c} - 2.06\times10^{-5}I_{BTA6D\_c} + (3.06\times10^{-4}+3.03\times10^{-2})I_{BTA3D\_c}$

$I_{BTA4D} = I_{BTA4D\_c} - 1.53\times10^{-3}I_{BTA6D\_c}$

$I_{BTA5D} = I_{BTA5D\_c} - 6.06\times10^{-2}I_{BTA6D\_c}$

$I_{BTA6D} = I_{BTA6D\_c} + (6.31\times10^{-10}+1.56\times10^{-7}+2.06\times10^{-5}+1.53\times10^{-3}+6.06\times10^{-2})I_{BTA6D\_c}$

The percentage of the deuterated analogues can be calculated:

$$\%BTAnD = \frac{I_{BTAnD}}{\sum_{k=1}^{6} I_{BTAkD}} \times 100\%$$

with $\sum_{k=1}^{6} I_{BTAkD}$ the sum of all intensities. The percentage of deuterated analogues was calculated for several time points and 3 independent samples and plotted as a function of time here.

7. References

(1) Lafleur, R. P. M.; Herziger, S.; Schoenmakers, S. M. C.; Keizer, A. D. A.; Jahzerah, J.; Thota, B. N. S.; Su, L.; Bomans, P. H. H.; Sommerdijk, N. A. J. M.; Palmans, A. R. A.; Haag, R.; Friedrich, H.; Böttcher, C.; Meijer, E. W. Supramolecular Double Helices from Small C3-Symmetrical Molecules Aggregated in Water. *J. Am. Chem. Soc.* **2020**, *142* (41), 17644–17652.

(2) Ludtke, S. J.; Baldwin, P. R.; Chiu, W. EMAN: Semiautomated Software for High-Resolution Single-Particle Reconstructions. *J. Struct. Biol.* **1999**, *128* (1), 82–97.

(3) Van Heel, M.; Harauz, G.; Orlova, E. V.; Schmidt, R.; Schatz, M. A New Generation of the IMAGIC Image Processing System. *J. Struct. Biol.* **1996**, *116* (1), 17–24.

(4) Lou, X.; Lafleur, R. P. M.; Leenders, C. M. A.; Schoenmakers, S. M. C.; Matsumoto, N. M.; Baker, M. B.; van Dongen, J. L. J.; Palmans, A. R. A.; Meijer, E. W. Dynamic Diversity of Synthetic Supramolecular Polymers in Water as Revealed by Hydrogen/Deuterium Exchange. *Nat. Commun.* **2017**, *8*, 15420.

(5) Balkenende, D. W. R.; Cantekin, S.; Duxbury, C. J.; Van Genderen, M. H. P.; Meijer, E. W.; Palmans, A. R. A. Enantioselective Synthesis of (R)-and (S)-1-2H-1-Octanol and Their Corresponding Amines. *Synth. Commun.* **2012**, *42* (4), 563–573.

(6) Leenders, C. M. A.; Albertazzi, L.; Mes, T.; Koenigs, M. M. E.; Palmans, A. R. A.; Meijer, E. W. Supramolecular Polymerization in Water Harnessing Both Hydrophobic Effects and Hydrogen Bond Formation. *Chem. Commun.* **2013**, *49* (19), 1963–1965.

(7) Leenders, C. M. A.; Baker, M. B.; Pijpers, I. A. B.; Lafleur, R. P. M.; Albertazzi, L.; Palmans, A. R. A.; Meijer, E. W. Supramolecular Polymerisation in Water; Elucidating the Role of Hydrophobic and Hydrogen-Bond Interactions. *Soft Matter* **2016**, *12* (11), 2887–2893.

(8) Max, J. J.; Chapados, C. Infrared Spectroscopy of Acetone-Methanol Liquid Mixtures: Hydrogen Bond Network. *J. Chem. Phys.* **2005**, *122* (1), 014504.

(9) Max, J. J.; Chapados, C. Infrared Spectroscopy of Acetone-Water Liquid Mixtures. I. Factor Analysis. *J. Chem. Phys.* **2003**, *119* (11), 5632–5643.

(10) Lou, X.; Schoenmakers, S. M. C.; van Dongen, J. L. J.; Garcia-Iglesias, M.; Casellas, N. M.; Fernández-Castaño Romera, M.; Sijbesma, R. P.; Meijer, E. W.; Palmans, A. R. A. Elucidating Dynamic Behavior of Synthetic Supramolecular Polymers in Water by Hydrogen/Deuterium Exchange Mass Spectrometry. *J. Polym. Sci.* **2021**, *59* (12), 1151–1161.
